# 

**Published:** 1882-03

**Authors:** 


CONTENTS.

| | |
|---|---|
| Men and Animals......107 | Self-Destroyers......130 |
| Diphtheria and its Cause......108 | Heart-Disease......133 |
| The Longevity of the Ancients......110 | Sleeping Together......134 |
| How to Behave at the Table......111 | The Instinct of Appetite......135 |
| Cure for Drunkenness......112 | The Achievements of Science......138 |
| A Lesson in Etiquette......113 | Health and Housekeeping......141 |
| Trust (poetry)......114 | Fever and Ague......144 |
| Tomatoes......114 | Cholera Certainties......149 |
| The Demon of Chloral......115 | Death's Weapons......153 |
| Bad Handwriting......116 | Laughter and Death (poetry)......155 |
| Savages' Notions of Nightmare......117 | Japan......155 |
| Silo Experience......119 | How to Travel Cheaply......158 |
| Burial Customs on the Continent......121 | A Small-Pox Preventive......160 |
| Cause of Death......123 | How Silks are Given Weight......162 |
| Spirits......125 | Daniel Webster's Presence......164 |
| To Stop Coughing......128 | Fishing on the Amazon......165 |

